# Development of an objective early detection model for depressive symptoms using voice emotion analysis technology: empirical prospective cohort study among call center operators

**DOI:** 10.1093/joccuh/uiaf060

**Published:** 2025-10-22

**Authors:** Naomichi Tani, Yoshihiro Takao, Sakihito Noro, Kazuki Sakai, Hisashi Eguchi, Takeshi Ebara

**Affiliations:** Department of Ergonomics, Institute of Industrial Ecological Sciences, University of Occupational and Environmental Health, Japan, 1-1 Iseigaoka, Yahatanishi-ku, Fukuoka, Kitakyushu 807-8555, Japan; ES Japan, Inc., 5F Dai-3 Kyoritsu Building, 2-50-9 Ikebukuro, Toshima-ku, Tokyo 171-0014, Japan; ES Japan, Inc., 5F Dai-3 Kyoritsu Building, 2-50-9 Ikebukuro, Toshima-ku, Tokyo 171-0014, Japan; Department of Ergonomics, Institute of Industrial Ecological Sciences, University of Occupational and Environmental Health, Japan, 1-1 Iseigaoka, Yahatanishi-ku, Fukuoka, Kitakyushu 807-8555, Japan; Department of Mental Health, Institute of Industrial Ecological Sciences, University of Occupational and Environmental Health, Japan, 1-1 Iseigaoka, Yahatanishi-ku, Fukuoka, Kitakyushu 807-8555, Japan; Department of Ergonomics, Institute of Industrial Ecological Sciences, University of Occupational and Environmental Health, Japan, 1-1 Iseigaoka, Yahatanishi-ku, Fukuoka, Kitakyushu 807-8555, Japan

**Keywords:** digital health technology, mental health, depression, voice and emotional analysis, voice analysis

## Abstract

**Objectives**: Voice and emotional analyses have gained attention in the diagnosis and monitoring of depression in clinical settings. However, evidence supporting its use for early detection in occupational health is lacking. This study aimed to develop a predictive model to identify early depressive symptoms in workers using voice and emotional analyses.

**Methods**: A prospective cohort study was conducted with 62 call center workers in Kumamoto Prefecture, Japan. The participants’ voices were automatically recorded during routine operations and analyzed using a voice and emotional analysis system based on Layered Voice Analysis. Depressive symptoms were assessed at 4 time points over 12 weeks using the Center for Epidemiologic Studies Depression Scale. Recursive Feature Elimination identified optimal voice features, while logistic regression was used to calculate the probability scores and build a predictive model for depressive symptoms. Predictive accuracy was evaluated using receiver operating characteristic curves and the area under the curve.

**Results**: The predictive model’s accuracy reached 0.783 (95% CI, 0.691-0.875) for the area under the curve, with a sensitivity of 0.649, a 1 − specificity of 0.174, and a cutoff value of 0.334. Individuals with composite voice indicators above the determined cutoff were significantly more likely to exhibit depressive symptoms 1 month later (odds ratio = 7.78; 95% CI, 3.27-18.5).

**Conclusions**: This study suggests that voice and emotional analysis can serve as an objective tool for the early identification of depressive symptoms in workplace settings. Accumulating real-world evidence from observational studies in diverse occupational populations is required to support broader implementation.

## Teaser

Key points:

- **What is already known on this topic:** Many studies have explored the use of voice emotion analysis for diagnosing and monitoring patients with depression, and its application is increasingly being integrated into clinical and therapeutic settings.
- **What this study adds:** Rather than focusing on diagnosis or monitoring, this study developed a predictive model that uses voice emotion analysis to forecast the early onset of depressive symptoms in working individuals.
- **How this study might affect research, practice, or policy:** This approach, which uses voice data to predict the onset of depressive symptoms 1 month in advance, may enable early alerts and proactive interventions for at-risk workers. For practical implementation, the legal and ethical handling of voice data as personal information must be clearly defined.

## 1. Introduction

Over the past 30 years, the global burden of disease due to mental disorders has continued to rise,^1^ with approximately 15% of the working population suffering from mental disorders.^2^ In particular, depressive disorders have been increasing in the working-age population over the age of 20.^1^ In 2019, they were estimated to account for 37.3% of the disability-adjusted life years (DALYs) lost due to mental disorders,^1^ which measure the burden of early death and disability.^1^^,^^3^ Projections indicate that depressive disorders will become the leading cause of DALYs among mental disorders by 2030.^4^ Thus, mental disorders are an issue in many countries worldwide, and Japan is no exception. There are 6.15 million patients with mental disorders in Japan, of whom approximately 1.69 million have depressive disorders, representing the largest category of mental disorders.^5^ Turning to Japanese workers, the Ministry of Health, Labour, and Welfare of Japan conducted the Special Survey on Industrial Safety and Health, which found that approximately 82.7% of workers reported psychological stress related to their current job or working life.^6^ Additionally, 13.5% of workplaces experienced worker absences of 1 month or more or resignations due to mental health problems in the past year.^6^ This highlights the urgent need for effective countermeasures against mental health disorders in Japan’s occupational health.

Researchers worldwide are investigating how digital health technologies (DHTs) can be applied to the treatment and management of mental disorders^7-10^—a field referred to as digital mental health (DMH). In the context of DMH, various types of data are used, including heart rate, physical activity, sleep, electroencephalogram, and voice and facial recognition.^11^ In recent years, research on emotion analysis using voice data has significantly increased in the field of DHTs.^12^ The fundamental theoretical process of voice and emotion analysis involves estimating changes in acoustic waveforms caused by variations at the phonatory and articulatory levels.^13^ These changes result from autonomic arousal and tension in striated musculature. The process also includes signal processing and pattern recognition to interpret emotional states and voice expressions using acoustic parameters such as fundamental frequency (F0), perturbation measures in the voice signal (jitter and shimmer), and spectral profiles (tilt, balance, distribution).^14^ The analysis of acoustic parameters in depressed patients yields characteristic acoustic waveforms,^15^^,^^16^ and this theoretical background has led to research on models for diagnosing and monitoring depression worldwide. Japanese studies have also examined the diagnosis of depression using voice emotion analysis.^17^^,^^18^

From a disease prevention perspective in the workplace, occupational health staff appear to be more interested in predicting the onset of depression than in diagnosing it. Workplace health care research needs to accumulate rigorous studies aimed at building a model for predicting depression, rather than diagnosing or monitoring it, in order to fill this gap. A previous study reported that early intervention within 1 month of the onset of acute stress disorder can prevent progression to more severe conditions.^19^ According to the “Manual for Implementing the Stress Check System Based on the Industrial Safety and Health Act,” published by the Ministry of Health, Labour, and Welfare, occupational health staff are required to respond to individuals with high stress levels within 1 month.^20^ Therefore, if the likelihood of developing mental health problems can be identified approximately 1 month in advance, occupational health staff can intervene earlier, potentially making a substantial contribution to the prevention of mental health disorders. Thus, this study aimed to develop an objective early detection model to predict depressive symptoms 1 month later using voice emotion analysis technology.

## 2. Methods

### 2.1. Study participants

This prospective short-term cohort study was conducted from July to October 2023 to determine whether workers’ voices could predict depressive symptoms. We recruited 240 call center operators employed at a company in Kumamoto Prefecture, Japan, whose duties included responding to complaints and inquiries received at the call center. Sixty-five workers aged 18-65 years responded to the survey. Eligibility criteria included individuals with at least 3 months of service, employed as regular, contract, or hourly workers, with no history of hospital visits for mental disorders, and who agreed to participate after receiving an explanation of the study.

This study was approved by the Institutional Review Board of the University of Occupational and Environmental Health, Japan (ID23-001, ID24-004). All participants provided written informed consent at enrollment.

### 2.2. Study design and data collection

**Figure 1:**
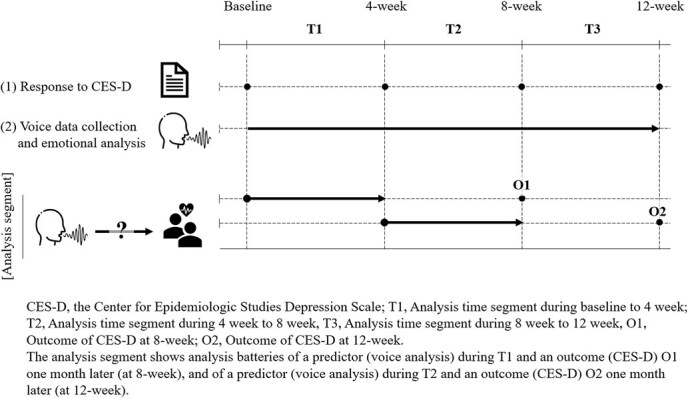
Scheme for building a model for predicting the occurrence of depressive symptoms by voice emotion analysis. Research question (RQ): Can voice emotion analysis predict the occurrence of depressive symptoms 1 month later? The prediction model for the Center for Epidemiologic Studies Depression Scale (CES-D) ≥16 at Outcome1 (O1) is validated on the voice data in periods baseline to 4 weeks (T1). Similarly, in periods 5 week to 8 weeks (T2), the prediction model for CES-D ≥ 16 at outcome 2 (O2) is validated.

An overview of this study is shown in Figure 1. This study used as the primary outcome the Center for Epidemiologic Studies Depression Scale (CES-D),^21^^,^^22^ a self-administered questionnaire assessing depressive symptoms, and participants’ voice data as a predictor in a prospective cohort design (details shown later). The follow-up period was 12 weeks, and participants were asked to complete the CES-D 4 times during this period (at baseline and at 4, 8, and 12 weeks). Voice recordings of customer interactions during daily call center operations were automatically collected and analyzed using a voice and emotion analysis system across 3 defined time segments: baseline to 4 weeks (T1), 5 to 8 weeks (T2), and 9 to 12 weeks (T3).

#### 2.2.1. Assessment of depressive symptoms as a primary outcome

We assessed depressive symptoms using the CES-D, which consists of 20 items, and evaluated depression based on the total score. For each item, respondents were asked to answer “no,” “1-2 days,” “3-4 days,” or “5 or more days” based on their experiences in the past week. The degree of depression was rated on a scale of 0 to 60, with higher scores indicating greater severity. A CES-D score of 16 or higher is considered indicative of depressive symptoms. We dummy-coded the CES-D score (1 = applicable, 0 = not applicable) based on whether it was 16 or higher, and used this coding as the outcome variable. The call center operators in this study represent occupations exposed to continuous stress due to emotional labor,^23^^,^^24^ with previous research showing that chronic and persistent mental distress and occupational stress progressively increase CES-D scores over time.^25^^,^^26^ The “Manual for Implementing the Stress Check System Based on the Industrial Safety and Health Act,” published by the Ministry of Health, Labour, and Welfare,^20^ recommends carrying out preventive interventions to workers with high stress levels within approximately 1 month of their examination. Therefore, we hypothesized that a predictive model could be developed by examining the association between voice data collected several weeks earlier and CES-D scores measured 1 month later, as an assessment of cumulative mental stress.

#### 2.2.2. Voice analysis data as a predictor

We used the Emotional Signature Analysis Solution (ESAS), based on the Layered Voice Analysis (LVA) system provided by ES Japan, Inc., to obtain objective voice parameters as predictors. LVA, the foundation of ESAS, analyzes voice fluctuations and speech variability to assess emotions, acute stress, and psychological distress, providing a visualization of an individual’s mental state using advanced algorithms.^27^ Academic validation of the LVA showed correlations between voice analysis results and State–Trait Anxiety Inventory anxiety scores, as well as between psychological agitation and systolic blood pressure, indicating its potential for assessing stress tolerance and psychological distress.^28^ In the occupational health field, the ESAS has also been used recently in mental health intervention studies.^23^

The participants’ speech was automatically recorded during call center operations and immediately processed by the ESAS’s proprietary algorithm, which converted the audio into 22 acoustic parameters (Table 1). These automatically generated acoustic parameters were exported as CSV files and provided directly to the university researchers for independent analysis. No employees of ES Japan participated in the analysis. From these acoustic parameters, a machine learning method, Recursive Feature Elimination (RFE),^29^ was used to extract variables associated with CES-D cutoff values (≥16). RFE is a type of Wrapper Method that identifies the optimal feature subset in a machine learning model. It builds a model using all the features and selects the optimal features by iteratively removing the least important features. We counted the outliers for each feature selected by RFE and used their counts as predictors. Outliers were defined as values above the cutoff for each parameter (Table 1). If the vendor did not recommend a cutoff value, the 75th percentile of the parameter was calculated from the dataset and used as the cutoff. For *x*_13_, which followed an approximately normal distribution, values exceeding plus 2 SDs above the mean were defined as outliers; for *x*_19_, all nonzero values were considered outliers. RFE was applied to voice data from T1 and CES-D data from the 4-week point. The 11 features selected by RFE in the T1 interval were fixed and applied unchanged to the T2 to 12-week prediction, thereby avoiding temporal leakage.

**Table 1:** Parameters of Emotional Signature Analysis Solution (ESAS), a voice emotion analysis system.

**Acoustic parameter**	**Summary**	**Min, max of parameter range**	**Cutoff value** ^**a**^
***x***_**1**_	Degree of psychological activity	0 to 100	≥40
***x***_**2**_	Balance between external load and internal response	0 to 100	≥30
***x***_**3**_	Balance between emotional and logical thinking	1 to 500	≥85
***x***_**4**_	Degree of concentration	0 to 100	—
***x***_**5**_	State of expectation	0 to 100	≥70
***x***_**6**_	Degree of elation	0 to 30	≥25
***x***_**7**_	Degree of indecision	0 to 30	—
***x***_**8**_	Degree of lack of confidence	0 to 30	—
***x***_**9**_	State of consideration	0 to 100	—
***x***_**10**_	Degree of recalling	0 to 30	≥10
***x***_**11**_	State of confusion	0 to 30	≥15
***x***_**12**_	Degree of interest	0 to 30	—
***x***_**13**_	Overall brain activity	0 to 100	—
***x***_**14**_	Level of self-confidence	0 to 30	—
***x***_**15**_	Parameters suggesting aggression	0 to 30	≥10
***x***_**16**_	Emotional parameters of the other person	0 to 100	—
***x***_**17**_	Possibility of some problems	0 to 100	—
***x***_**18**_	Auxiliary indicators	±100	—
***x***_**19**_	Dissatisfaction or sadness	0 to 30	—
***x***_**20**_	Satisfaction or joy	0 to 30	—
***x***_**21**_	High stress state	0 to 30	≥20
***x***_**22**_	Degree of general emotional movement	0 to 30	≥20

a Parameters recommended by the vendor.

### 2.3. Prediction model and statistical analysis

#### 2.3.1. Primary analyses

We used 4 weeks of audio data from T1 and T2, along with CES-D response data at 8 weeks and 12 weeks, to develop a model predicting depressive symptoms 1 month later. Data batteries at the 8-week CES-D corresponding to T1 and at the 12-week CES-D corresponding to T2 were used for analysis as vertically stacked longitudinal datasets (Figure 1). The probability score was calculated using multivariable logistic regression, with the dichotomized CES-D as the dependent variable, the number of outliers for each of the 11 variables selected by RFE as independent variables, according to the following formula:


(1) \begin{equation*} Probability\ score=\frac{1}{1+{\mathit{\exp}}^{-\left({\beta}_0+{\beta}_1{X}_1+\cdots +{\beta}_{11}{X}_{11}\right)}} \end{equation*}

Using this calculation, we attempted to obtain a synthetic variable (an integrated predictor) representing the probability of exposure to a predictor of depressive symptoms, and coefficients (β) for each covariate. In this study, the probability score served as an intermediate variable derived from voice features, rather than a direct model of the raw acoustic parameters.

The correlation between the CES-D score and the synthetic variable was analyzed using the Pearson correlation coefficient model, demonstrating that the synthetic variable from the probability score is a valid index for assessing depression. Furthermore, a model was constructed using the dummy-coded CES-D as the outcome variable and the synthetic variable from the probability score (SVPS) as the integrated predictor. Receiver operating characteristic (ROC) curves were drawn to determine the cutoff values of the model, and their accuracy was assessed using the area under the curve (AUC). Odds ratios (ORs) and 95% CIs were calculated using the dummy-coded CES-D as the outcome variable and the cutoff value of SVPS (COSVPS) (above vs below the cutoff) as a predictor.

#### 2.3.2. Sensitivity analyses

We conducted a sensitivity analysis to evaluate the generalizability and robustness of the predictive model. Time-split pseudo-external validation assessed whether model performance could be maintained across intervals by calculating the AUC, sensitivity, specificity, and OR of the COSVPS using the cutoff value derived from Equation 1 on data separated into T1 with outcomes at Week 8 (T1-8 wk) and T2 with outcomes at Week 12 (T2-12 wk). Furthermore, to reduce the risk of underestimating SEs due to within-participant correlations, we performed a generalized estimating equations (GEE) model that accounted for both participant clustering and the interaction between COSVPS and time intervals (T1, T2), thereby assessing the robustness of the predictive model derived from the main analysis.

All analyses were conducted using Python version 3.11.5, and R version 4.3.1 (R Foundation for Statistical Computing, Vienna, Austria). The statistical significance level was set at *P* < .05.

## 3. Results

The demographic characteristics of the participants at baseline are presented in Table 2. After excluding participants with missing voice data, 62 were included in the analysis. The mean age of the participants was 45.2 years; 88.7% were female, 46.8% belonged to Section 1, 50% were employed full-time, 29% were part-time, and 21% were employed as dispatched workers.

**Table 2:** Demographic characteristics of participants (*n* = 62).

	**Prevalence,** ^**a**^ ***n* (%)**		**Depressive symptoms**		
		**Overall (*n* = 62)**	**Negative (*n* = 43)**	**Positive (*n* = 19)**	***P* value**
**Age, mean (SD), y**		45.2 (14.6)	46.2 (15.0)	42.8 (14.0)	.408
**Sex, *n* (%)**					.665
**Male**		7 (11.3)	4 (9.3)	3 (15.8)	
**Female**		55 (88.7)	39 (90.7)	16 (84.2)	
**Department,** ^**b**^ ***n* (%)**					.168
**Section 1**		29 (46.8)	23 (53.5)	6 (31.6)	
**Section 2**		33 (53.2)	20 (46.5)	13 (68.4)	
**Employment status, *n* (%)**					.370
**Full-time**		31 (50.0)	22 (51.2)	9 (47.4)	
**Part-time**		18 (29.0)	14 (32.6)	4 (21.1)	
**Dispatched**		13 (21.0)	7 (16.3)	6 (31.6)	
**CES-D,** ^**c**^ **median (range)**					
**Baseline**	19 (30.6)	12.0 (1-31)	9.0 (1-15)	21.0 (17-31)	<.001
**4 weeks**	24 (38.7)	13.0 (0-40)	9.0 (0-37)	22.0 (6-40)	<.001
**8 weeks**	18 (29.0)	9.5 (0-44)	8.0 (0-29)	20.0 (6-44)	<.001
**12 weeks**	19 (30.6)	12.0 (0-33)	10.0 (0-29)	18.5 (9-33)	<.001

Abbreviation: CES-D, Center for Epidemiologic Studies Depression Scale.

a Prevalence of depressive symptoms was defined as a CES-D score ≥16.

b The call center operators in Sections 1 and 2 have different procedures, because of the different companies with which they are contracted.

c At baseline, participants with CES-D scores ≥16 were classified as the positive group; those with lower scores were classified as the negative group.

**Figure 2:**
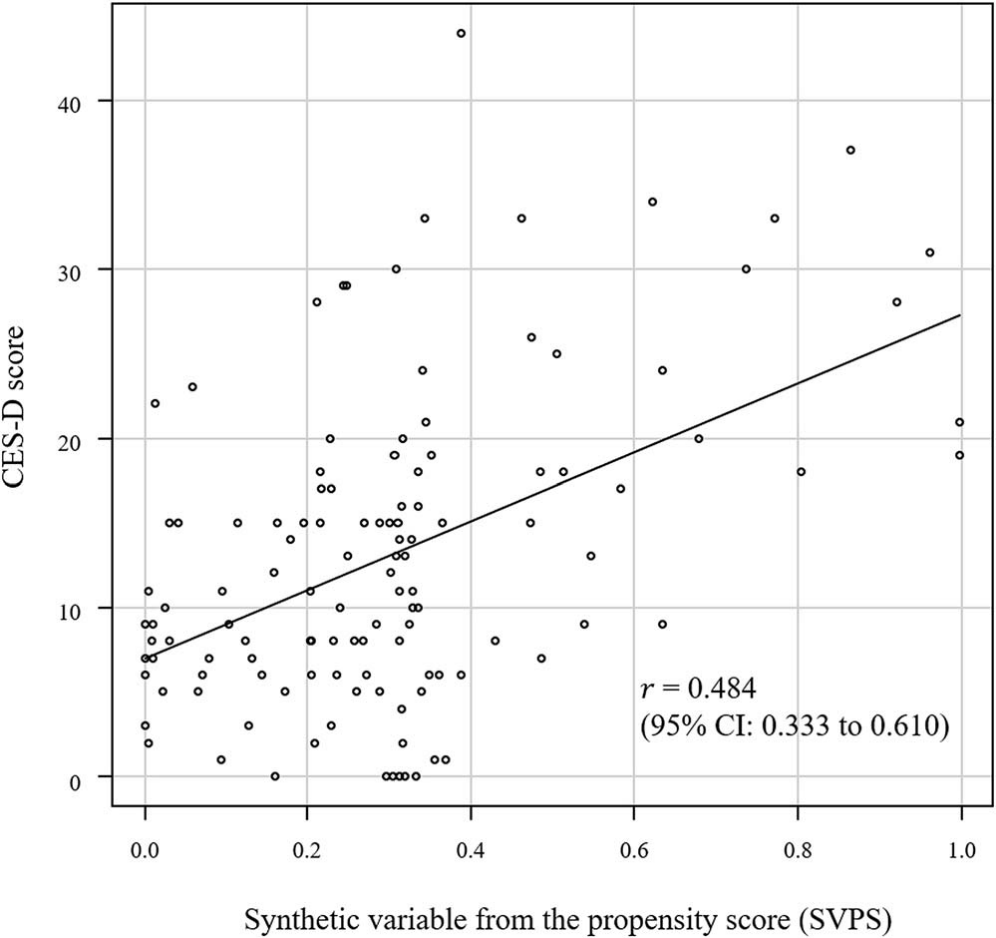
The scatter plot of the CES-D score and the SVPS in participants. Abbreviations: CES-D, Center for Epidemiologic Studies Depression Scale; SVPS, synthetic variable from the probability score.

The following 12 variables were extracted as variables associated with the CES-D as a result of the RFE analysis: *x*_2_, *x*_3_, *x*_4_, *x*_6_, *x*_8_, *x*_10_, *x*_11_, *x*_13_, *x*_14_, *x*_16,_  *x*_17_, and *x*_19_. However, *x*_16_, a parameter for customers who called call centers, was excluded because it was inappropriate as a variable. Therefore, using the 11 variables, the probability score for the integrated predictor was calculated by multivariate logistic regression analysis using Equation 2. Figure 2 shows a scatter plot of the CES-D score and SVPS using the Pearson correlation coefficient model. A significant moderate correlation was obtained (𝑟 = 0.484; *P* < .001; 95% CI, 0.333-0.610).


(2) \begin{align*} &Probability\ Score\notag \\ &=\frac{1}{1+{\mathit{\exp}}^{\begin{array}{c}\Big(0.76960223+0.00009187{x}_2-0.00018121{x}_3\\-0.0001199{x}_4+0.00057643{x}_6 {}-0.0002568{x}_8\\+0.00008715{x}_{10}-0.00183546{x}_{11}+0.00044089{x}_{13}\\+0.00017764{x}_{14} {}-0.00008483{x}_{17}-0.00057656{x}_{19}\Big)\end{array}}} \end{align*}

**Figure 3:**
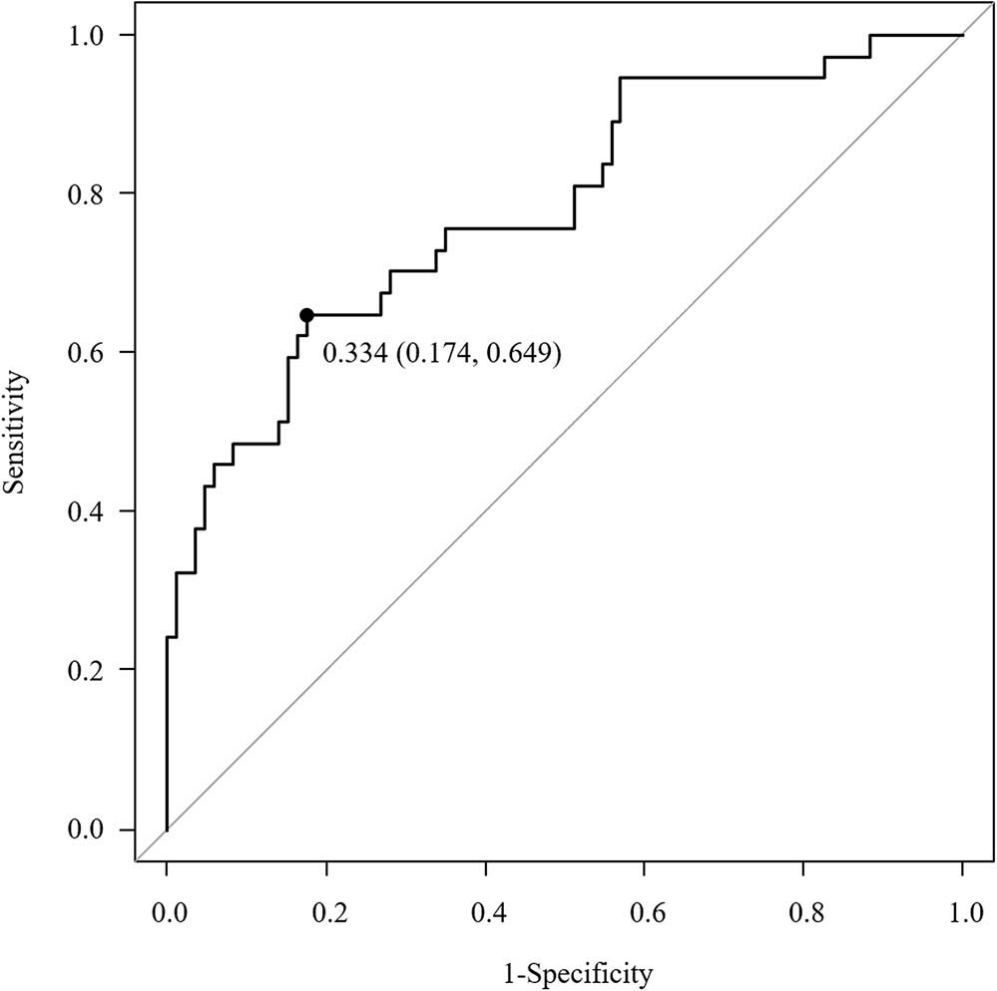
The receiver operating characteristic (ROC) curves for depression symptom prediction model.

We constructed a predictive model of depressive symptoms using the SVPS. Our model’s ability to distinguish depressed from nondepressed participants was quantified by ROC analysis. The ROC curve in Figure 3 shows the classifier’s performance on the training set over a range of model values (AUC: 0.783; 95% CI, 0.691-0.875). The cutoff value for predicting depressive symptoms was 0.334, achieving a predictive performance (sensitivity, 0.649; 1 − specificity, 0.174). The SVPS was dichotomizing at a cutoff value of 0.334 (ie, COSVPS), and the resulting analysis showed that the OR for depressive symptoms was 7.78 (95% CI, 3.27-18.5) for values ≥0.334 compared with values <0.334 (*P* < .001). The depression indicator is given by Equation 3:


(3) \begin{equation*} Prediction\ model=\frac{1}{1+{\mathit{\exp}}^{\left(1.624-2.051\times COSVPS\right)}} \end{equation*}

The results of the sensitivity analyses are shown in Table 3. A GEE model confirmed the robustness of the predictive model, showing that participants with COSVPS ≥0.334 had significantly greater odds of depressive symptoms than those with COSVPS <0.334 (OR = 5.18; 95% CI, 1.59-16.9; *P* < .01). The AUC values obtained using time-split pseudo-external validation were 0.760 (95% CI, 0.619-0.901) for T1-8 wk and 0.838 (95% CI, 0.731-0.946) for T2-12 wk, which were close to those of the main predictive model. Table S1 presents the robustness of the outlier threshold (75th percentile) used for features without vendor-recommended thresholds.

**Table 3:** Evaluation of robustness and generalizability of the prediction model through sensitivity analysis.

	**OR (95% CI)** ^**a**^	**AUC (95% CI)**	**Sensitivity**	**1 − specificity**
**GEE model**	5.18 (1.59 to 16.9)^**^			
**Time-split pseudo-external validation** ^**b**^				
T1-8 wk	3.23 (1.03 to 10.1)^*^	0.76 (0.62 to 0.90)	0.56	0.28
T2-12 wk	11.1 (3.16 to 39.3)^**^	0.83 (0.73 to 0.95)	0.68	0.16

Abbreviations: AUC, area under the curve; GEE, generalized estimating equations; OR, odds ratio.

a Statistical significance denoted by: ^*^P < .05; ^**^P < .01.

b In the time-split pseudo-external validation, the predictive model’s performance was evaluated using the cutoff value (0.334) for data divided into T1 and outcomes at Week 8 (T1-8 wk), and T2 and outcomes at Week 12 (T2-12 wk).

## 4. Discussion

To our knowledge, this study is the first to demonstrate the potential of a predictive model for depression symptoms in call center operators 1 month later using voice emotion analysis parameters. The SVPS score, an integrated predictor extracted from 11 acoustic parameters selected by machine learning, correlated moderately positively with the participants’ CES-D scores. Furthermore, the AUC of the model predicting depressive symptoms using the COSVPS was 0.783, indicating moderate accuracy, and sensitivity analyses confirmed a certain degree of robustness and generalizability of the model. A previous study reported an accuracy rate of 69.5% for a model that determined depression severity based on speech.^30^ Another previous study compared the accuracy of a machine learning model and a logistic regression model in classifying depressive symptoms from speech, and reported that the logistic regression model was more accurate, with accuracies of 75.0% for females and 81.8% for males.^31^ The accuracy of our predictive model is generally similar to the results of these studies. However, previous studies in Japan have shown that a diagnostic system using the voice of patients with major depression achieved an AUC as high as 0.97, which is notably high.^18^ Although our model is less accurate than this previous report, this may be because the model predicts 1 month in advance and uses healthy workers, rather than clinically diagnosed patients, as participants. In practice, if a higher false-positive rate is acceptable, using a model with greater sensitivity may offer predictive potential for early detection of depressive symptoms in occupational health settings.

A systematic review highlighted several advantages of voice and emotion analysis, including improved accessibility to mental health assessment and treatment, applicability across a wide range of psychiatric conditions beyond depression, and the feasibility of remote monitoring irrespective of time or location.^32^ It also summarized both the promise and the methodological challenges of this emerging field. In the context of prevention, König et al^33^ reported that voice analysis conducted during telephone interviews could quantify stress levels in health care workers during the COVID-19 pandemic, underscoring the feasibility of remote stress monitoring. Building on these advances, our study contributes a novel dimension: the prospective prediction of depressive symptoms 1 month in advance, rather than contemporaneous assessment. This approach is consistent with occupational health intervention timelines, such as Japan’s occupational Stress Check Program, and extends the utility of voice and emotion analysis from diagnostic applications to proactive intervention planning. Moreover, recent progress in machine learning—including techniques robust to noise and device variability—suggests that accurate speech-based mental health prediction may soon be deployable on virtually any device,^34^ further broadening its real-world applicability.

Although the use of DHT in mental health prevention and occupational health activities in Japan is limited, occupational health staff have shown interest in how such technology can be applied.^35^ Therefore, it is necessary to explore how such technologies can be applied in preventive activities.^12^ For example, voice emotion analysis could be used as an add-on feature to online conferencing systems, which have been used with increasing frequency since COVID-19, to determine stress reactions in real time. Other possibilities include using digital avatars equipped with artificial intelligence (AI) and voice emotion analysis to conduct occupational health interviews with workers, enabling the detection of stress reactions regardless of location and alerting occupational health staff when the risk of depressive symptoms increases. Increased research on occupational health applications of voice emotion analysis could help realize these possibilities.

However, voice and emotion analyses, the focus of this study, have not been included in previous systematic reviews of intervention studies.^35^ In other words, there is currently a lack of evidence regarding the application of voice-emotion analysis in the field of occupational health, and its effectiveness remains unclear. To facilitate the application of DHTs (including voice and emotion analysis) in Japanese occupational health settings, it is necessary to establish a system for evaluating the quality standards of DHT products. However, Japan has yet to establish clear criteria for evaluating the evidence supporting DHTs.

In contrast, the National Institute for Health and Care Excellence (NICE) in the United Kingdom developed the Evidence Standards Framework (ESF) to classify DHTs into 3 tiers (A to C) based on their potential risk and impact on clinical outcomes.^36^^,^^37^ Tier A includes low-risk tools, such as wellness apps and step counters, which require only minimal evidence. Tier B includes tools for managing mild health conditions that require moderate evidence, such as observational studies. Tier C comprises high-risk technologies, including AI diagnostic tools and digital therapeutics, which require robust evidence from randomized controlled trials (RCTs). This framework may serve as a valuable reference for establishing evidence-based standards for DHTs in Japan. However, conducting large-scale RCTs (Tier C) among workers is challenging. Therefore, it is necessary to assess the risks and effectiveness of DHT services, such as voice and emotion analyses, by accumulating Tier B-level evidence, including observational studies.

Another major barrier to implementing speech emotion analysis in occupational health is the lack of clear regulations on handling voice data under Japan’s Personal Information Protection Law.^24^ In contrast, in the European Union (EU), voice is classified as biometrically identifiable data—alongside facial features, eye movements, and gait—and is therefore subject to the General Data Protection Regulation (GDPR), which restricts its transfer outside the European Economic Area.^38^^,^^39^ Moreover, EU regulations generally prohibit AI-based emotion estimation for applications other than health care–related products.^38^ Privacy and ethical concerns regarding these sensitive personal data arise because continuous monitoring of workers’ actions, coupled with the reporting of such activities to employers or managers, could result in reprimands or penalties, thereby raising significant issues regarding individual autonomy and workplace fairness.^40^ Addressing these concerns and uncertainties through standardized evaluation frameworks for DHTs and legal guidelines could facilitate the safe and ethical adoption of these DHTs, including voice-emotional analysis.

This study has several limitations. First, the relatively small sample size may have influenced the results and limited statistical power. Second, selection bias is a concern, as the study population consisted exclusively of call center operators; thus, the predictive model requires validation in workers from other occupational settings. Moreover, the fact that only 62 of the 240 eligible participants were included in the analysis further underscores the potential for selection bias. Third, there are limitations in estimating the emotions of workers who have little or no verbal interaction, such as assembly line workers, long-haul truck drivers, and cleaning staff. Moreover, although the time-split pseudo-external validation provided evidence of generalizability, it was conducted within the same cohort, and formal external validation is still lacking. In addition, further validation using bootstrap methods to correct for potential optimism bias would strengthen confidence in the model’s robustness. Therefore, the generalizability of the findings should be interpreted with caution. Finally, ESAS has been described as a technology that, through LVA, captures minute changes in speech waveforms and estimates the speaker’s emotions regardless of the social position of the interlocutor or the conversational context. However, although ESAS is based on LVA technology, its accuracy in detecting psychological stress has not been fully established. In addition, ESAS immediately converts the input speech into 22 parameters via its proprietary automatic algorithm, and the original voice waveforms cannot be accessed by researchers. This black-box processing limits the ability to verify technical validity or apply alternative analytical methods.

## 5. Conclusions

The findings of this study suggest that voice emotion analysis holds potential for the objective early detection of workers’ depressive symptoms 1 month in advance and may be applicable in the field of occupational health. It is important to build a body of evidence through observational studies using real-world data from a variety of occupations to evaluate its effectiveness further.

## Supplementary Material

Web_Material_uiaf060

## Data Availability

Data used in this study will be shared by the corresponding author upon request.
