# Supplementary material for: Development of an objective early detection model for depressive symptoms using voice emotion analysis technology: empirical prospective cohort study among call center operators
Source: J Occup Health. 2025 Oct 22;67(1):uiaf060. doi: 10.1093/joccuh/uiaf060 (PMC12662643; doi:10.1093/joccuh/uiaf060)
Supplement: Web_Material_uiaf060 [file web_material_uiaf060.docx]

| Supplementary Table 1. Robustness of outlier thresholds ^a^. | | | | | |
| --- | --- | --- | --- | --- | --- |
|  | Cut-off value | OR(95%CI) | AUC(95%CI) | Sensitivity | 1-Specificity |
| 70th percentile | 0.322 | 6.51 (2.79 to 15.2) *** | 0.78 (0.69 to 0.87) | 0.68 | 0.22 |
| 80th percentile | 0.334 | 7.78 (3.27 to 18.5) *** | 0.78 (0.69 to 0.88) | 0.65 | 0.17 |
| Abbreviation: OR, Odds ratio; CI, Confidence interval; AUC, Area under the curve; *** *p* <.001. ^a^ For parameters without vendor-recommended thresholds, we redefined thresholds at 70% and 80% to calculate Cut-off values, ORs, AUCs, Sensitivity, and Specificity, thereby verifying robustness at the 75th percentile. | | | | | |
